# Supplementary material for: Constraint Release Rouse Mechanisms in Bidisperse Linear Polymers: Investigation of the Release Time of a Short-Long Entanglement
Source: Polymers (Basel). 2023 Mar 21;15(6):1569. doi: 10.3390/polym15061569 (PMC10058622; doi:10.3390/polym15061569)
Supplement: Supplementary file 1 [file polymers-15-01569-s001.zip › polymers-2226069-supplementary.pdf]

polymers

MDPI

Supplementary Information: Investigation of the Release Time of a Short-Long Entanglement

Céline Hannecart [1,2], Christian Clasen [2] and Evelyne Van Ruymbeke [1,*]

[1] Bio- and Soft Matter, Institute of Condensed Matter and Nanosciences, Université catholique de Louvain, 1348 Louvain-la-Neuve, Belgium
[2] Soft Matter, Rheology and Technology, Department of Chemical Engineering, KU Leuven, 3001 Leuven, Belgium
* Correspondence: evelyne.vanruymbeke@uclouvain.be

1. Viscoelastic Properties Under iso-T_g Conditions

The linear viscoelastic data coming from the literature and presented at a specific temperature were horizontally shifted in order to correspond to iso-T_g conditions ($T_{ref} - T_g$). The validity of the shifting can be verified in Figure S1 for the monodisperse PS matrices from ref. [1]: while the data of the samples with $M_c < 70 kg.mol^{-1}$ do not superimpose at high frequencies when they are plotted at the same reference temperature $T_{data} = 167\,°C$ (dashed grey lines), a good superposition is obtained under iso-T_g conditions (symbols).

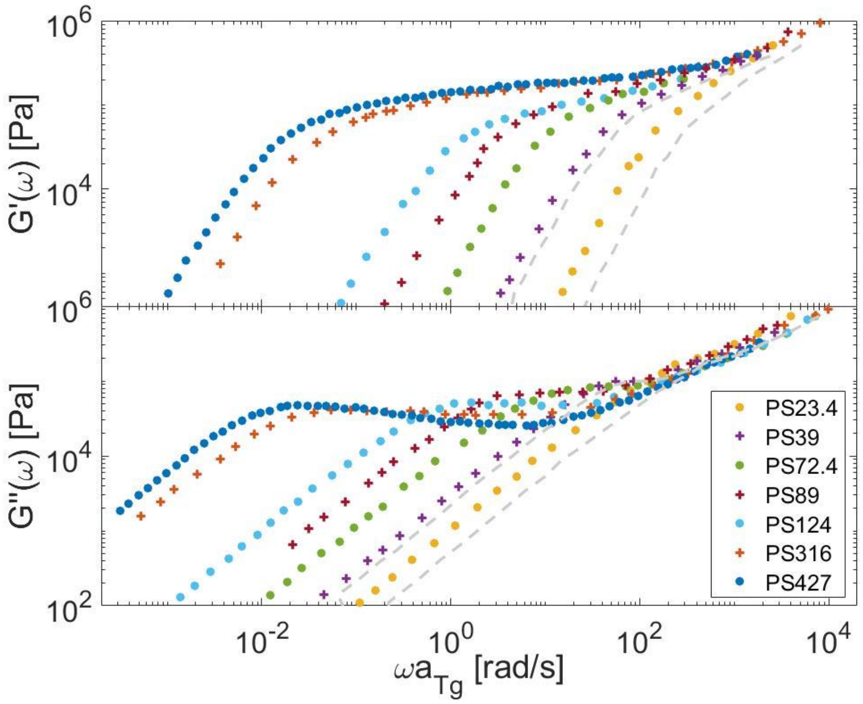

Figure S1. Experimental storage and loss moduli of monodisperse PS samples [1] plotted under iso-T_g conditions ($T_{ref} - T_g = 60.4$, symbols) and at $T_{data} = 167$ °C (dashed grey lines).

Then, the experimental data of the blends PS316-39 have been shifted to iso-T_g, based on the Fox-Flory equation and considering that the glass transition temperature of PS39 is around 103.8 °C. To do so, new reference temperatures have been used for each blend: T_{ref} = 164.2, 164.3, 164.3 and 164.5 °C, leading to a_{Tg} = 0.725, 0.735, 0.741, and 0.752 for ν_L = 0, 3, 5, and 10%, respectively.

Figure S2 presents the shifted data and shows a good superposition of the relaxation moduli at high frequencies after correction of the reference temperature. This confirms the calculated values of T_g and the corresponding shift factors.

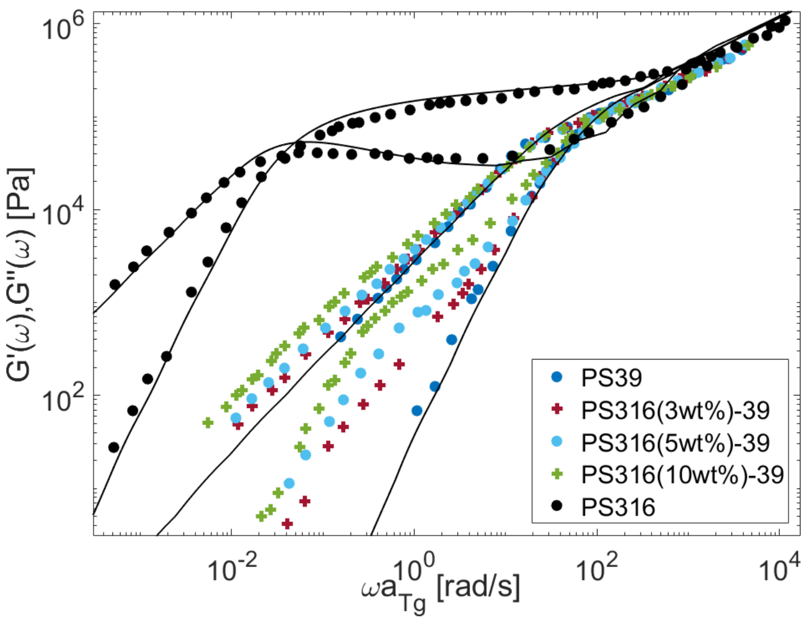

Figure S2. Shifted storage and loss moduli from reference [1] of PS316-39 blends (symbols), compared to the predictions obtained with the TMA model for the monodisperse components PS39 and PS316 (black curves).

2. Universality of the Constraint Release Rouse Process in Bidisperse Linear Blends with Self-Unentangled Long Chains

As discussed in the main paper, assuming that the CRR time of the long chains is proportional to $Z_S^{2.5}Z_L^2$ (with Z_S and Z_L being the number of entanglement segments along the short and the long chains, respectively), this leads to a universal behavior of this process for the different chemistries tested in the present work (PS, PI, PBD, and PMMA).

Since the blends PS2810-72.4 [2] and PI626-17.6 [3] have the particularity to have both the same Z_S and Z_L, according to this universality concept, their storage modulus normalized by their different plateau modulus, G_N^0, and relaxation time of an entanglement segment, τ_e, should completely superimpose if the weight fraction of long and short chains are the same. However, their proportion of long chains differs, with $\upsilon_L = 0.01$ in the blend PS2810-72.4, and $\upsilon_L = 0.005$ in the blend PI626-17.6. Accounting for this difference, it is expected, if we assume the universality of the CRR process, that the terminal regime of the normalized storage moduli $\frac{G'}{\upsilon_L G_N^0}$ as a function of $\omega\tau_e$ superimpose, as well as that the normalized storage moduli $\frac{G'}{G_N^0}$ as a function of $\omega\tau_e$ superimpose at high and intermediate frequencies (as the proportion of long chains is negligible). These two equivalences are confirmed in Figure S3.

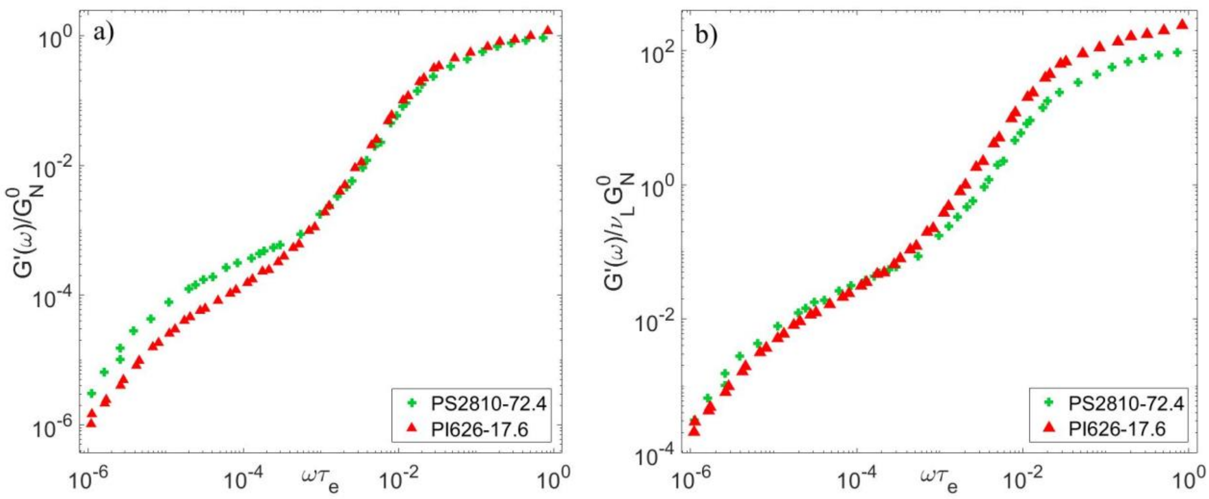

Figure S3. (**a**) Storage modulus data of PS2810-72.4 and PI626-17.6 normalized by (**a**) G_N^0, or (**b**) $G_N^0 \nu_L$, with respect to $\omega\tau_e$.

3. Non-Rouse Feature of the CR Dynamics

While the viscoelastic relaxation of a dilute long chain in a bidisperse blend is well-described by a CR-Rouse process, Figure 8 of the main paper exhibits some deviation between the normalized long chain storage modulus data and a pure Rouse-like relaxation at high frequencies, as was already noticed by Sawada et al. [3] for PI/PI dilute blends. This deviation can be explained by the non-Rouse feature of the CR dynamics, observed by Watanabe et al. [4] when investigating the dielectric relaxation of dilute dipole-inverted PI blends. Indeed, the authors showed that the eigenfunctions related to the long probe chain motion are distorted from the perfect sinusoids expected for Rouse-like chain relaxation, especially for high-order modes.

In addition to the possible effect of tension re-equilibration on the relaxation of long chains [4], the non-Rouse feature of the CR mechanism is also due to the collapse of fast CRR modes, as proposed by Sawada et al. [3]. Indeed, by noticing that local CR jumps are activated by the matrix motions, they concluded that CRR relaxation modes cannot happen on a timescale smaller than $\tau_{d,S}$, the final relaxation time of the matrix chains. Therefore, we take this condition into account in the model by considering that the relaxation time of a CRR relaxation mode p can never be smaller than $\tau_{d,S}$:

$$\tau_p = \frac{\tau_{CRR,L}}{p^2} \geq \tau_{d,S} \quad \text{(S1)}$$

Therefore:

$$G_{CRR,L}(t) = \frac{\nu_L \rho RT}{M_L} \sum_{p=1}^{Z_L} e^{-2t/\tau_p} \quad , \quad \tau_p = \frac{\tau_{CRR,L}}{p^2} \geq \tau_{d,S} \quad \text{(S2)}$$

Results obtained with Equations S1 and S2 are compared to the experimental data in Figure S4, which show the long chain contributions to the storage moduli of the PI blends [3]. The results obtained by considering the pure CRR process (Equation 7) in the main paper) are also shown (dashed curves). A very good agreement is found with Equation S2, which confirms the idea that CR jumps cannot happen on a timescale smaller than $\tau_{d,S}$, explaining, in part, the observed non-Rouse feature of the CR mechanism.

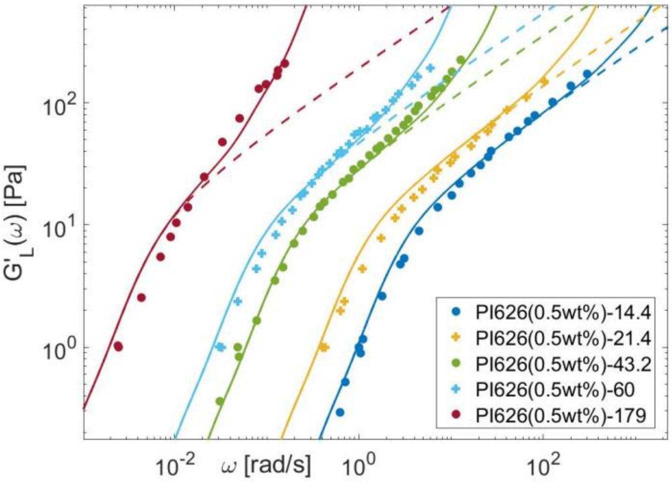

Figure S4. Comparison between experimental (symbols) viscoelastic storage modulus data of PI626 blends in different matrices with a modified CRR relaxation modeled by Equation S2 (continuous curves) or with a pure CRR mechanism (dashed curves).

Since this deviation happens on a timescale of the order of $\tau_{d,S}$, its effect on the viscoelastic relaxation modulus of the whole blend is negligible; on this timescale, G_{blend} is largely dominated by the relaxation of the short matrix chain, concealing the contribution of the long matrix chain to the viscoelastic relaxation modulus. Therefore, neglecting the deviation from a pure CRR mechanism is a reasonable approximation when modeling the viscoelastic relaxation of a dilute bidisperse blend.

Reference

1. Watanabe, H.; Sakamoto, T.; Kotaka, T. Viscoelastic Properties of Binary Blends of Narrow Molecular Weight Distribution Polystyrenes. 2. *Macromolecules* **1985,** 18, 1008-1015.
2. Watanabe, H.; Sakamoto, T.; Kotaka, T. Entanglements in Linear Polystyrenes. *Macromolecules* **1985,** 18, 1436-1442.
3. Sawada, T.; Qiao, X.; Watanabe, H. Viscoelastic Relaxation of linear polyisoprenes: Examination of constraint release mechanism. *Nihon Reoroji Gakkaishi* **2006,** 35, 1, 11-20.
4. Watanabe, H.; Urakawa, O.; Kotaka, T. Slow Dielectric Relaxation of Entangled Linear cis-Polyisoprenes with Asymmetrically Inverted Dipoles. 2. Behavior in a Short Matrix, *Macromolecules*. **1994,** 27, 3525-3536.
